# An approach to adjust standardized mortality ratios for competing cause of death in cohort studies

**DOI:** 10.1007/s00420-015-1097-z

**Published:** 2015-10-19

**Authors:** Matthias Möhner

**Affiliations:** Federal Institute for Occupational Safety and Health (BAuA), Berlin, Germany

**Keywords:** Cohort study, Lung cancer, Pneumoconiosis, Coal miners, Standardized mortality ratio, Adjustment

## Abstract

**Purpose:**

The calculation of standardized mortality ratios (SMRs) is a standard tool for the estimation of health risks in occupational epidemiology. An increasing number of studies deal with the analysis of the mortality in employees suffering from an occupational disease like silicosis or coal-worker pneumoconiosis (CWP). Their focus lies not on the mortality risk due to the occupational disease itself, but on other diseases such as lung cancer or heart diseases. Using population-based reference rates in these studies can cause misleading results because mortality rates of the general population do not reflect the elevated mortality due to the occupational disease investigated. Hence, the purpose of the present paper is to develop an approach to adjust the risk estimates for other causes of death with respect to the effect of an occupational disease as a competing cause of death in occupational mortality cohort studies.

**Methods:**

To overcome the problems associated with SMRs, the paper makes use of proportional mortality ratios (PMR), which are a further approach for the estimation of health risks in occupational epidemiology. The cause-specific SMR can be rewritten as a product of PMR times the overall SMR. The PMR can be adjusted by ignoring the competing cause of death. Hence, an adjusted cause-specific SMR can be derived by multiplying this adjusted PMR with the overall SMR. This approach is applied to studies concerning lung cancer risk in coal miners suffering from CWP.

**Results and conclusions:**

The usual approach for calculating SMRs leads to an underestimation of the real lung cancer risk in subgroups of miners suffering from CWP. The same effect can be observed in workers exposed to respirable silica already suffering from silicosis. The presented approach results in more realistic risk estimation in mortality cohort studies of employees suffering from an occupational disease. It is easily calculable on the basis of usually published values of observed cases and the corresponding cause-specific SMR.

## Introduction

It is well known that occupational exposures to crystalline silica and coal mine dust can cause nonmalignant respiratory diseases (NMRD), especially silicosis and coal-worker pneumoconiosis (CWP) (Miller and MacCalman 2010). Moreover, silica dust is classified as a group 1 carcinogen (sufficient evidence) by the International Agency for Research on Cancer (IARC). However, the lung cancer risk in coal miners is under discussion. While two cohort studies based on incidence data indicate an elevated lung cancer risk in miners suffering from CWP (Morfeld et al. 2005; Tomaskova et al. 2012), studies based on mortality data and looking at standardized mortality ratios (SMRs) only do not give similar risk estimates. The SMRs are based almost always on reference rates of the general population and do not take into account the elevated mortality from NMRD, especially from CWP. Therefore, the aim of the present paper is to find a practicable approach to estimate the mortality for a certain cause of death, taking into account that all cohort members suffer from another disease, which acts as a competing cause of death.

The effect of this adjustment on the risk estimators will be demonstrated for various cohort studies on miners. However, a review of a potential causal link between CWP and lung cancer is beyond the scope of this paper.

## Methods

The SMR is by far the most frequently used statistical measure for the analysis of the effect of risk factors on health in occupational cohort studies. The knowledge of the population-at-risk by age, sex and race as well as corresponding rates for the reference population is necessary for the calculation of SMRs. A further useful measure is the proportional mortality ratio (PMR), which compares the proportion of all deaths due to a specific cause in the cohort to the corresponding proportion in a comparison group. This measure is mainly used if the mortality in the reference population is described by the number of deaths without any further characteristics of this population. The disadvantage of the PMR is that it does not give information on the total force of mortality. Decouflé and colleagues have discussed the general relationship between SMR and PMR (Decouflé et al. 1980). They have shown that a rough approximation of cause-specific SMR can be derived by multiplying the cause-specific PMR with the overall SMR.

The ratio between the cause-specific SMR and the overall SMR, also known as the relative standardized mortality ratio (RSMR), illustrates the share of a single cause of death in the overall mortality. The RSMR also describes the ratio of relative frequencies for the cause *i* between the cohort under investigation and the reference population, taking into account the age structure in the cohort.

Some general notations are given in Table 1 to elucidate the method proposed as simple as possible. Thus, the relationship between PMR and SMR can be described by the following equation (cf. Eq. 5 in Decouflé et al. 1980):

1 $${\text{RSMR}}_{i} = \frac{{{\text{SMR}}_{i} }}{\text{SMR}} = \frac{{\frac{{{\text{OBS}}_{i} }}{\text{OBS}}}}{{\frac{{{\text{EXP}}_{i} }}{\text{EXP}}}} = {\text{PMR}}_{i}$$

**Table 1:** Notations for statistical quantities discussed in text

Quantity	Study cohort	Expected values*
Total deaths (all causes)	OBS	EXP
Deaths from the specific cause of interest	OBS_*i*_	EXP_*i*_
Deaths from competing cause	OBS_0_	EXP_0_
Overall standardized mortality ratio	SMR = OBS/EXP	
Standardized mortality ratio for cause *i*	SMR_*i*_ = OBS_*i*_/EXP_*i*_	
Relative standardized mortality ratio for cause *i*	RSMR_*i*_ = SMR_*i*_/SMR	

* Calculated using a set of age-specific rates from the reference population

Strictly speaking, the last equation holds only without any adjustment for confounding factors like age. But several studies have shown empirically that the age-standardized ratio between SMR_*i*_ and PMR_*i*_ closely approximates the age-standardized overall SMR (Decouflé et al. 1980; Kupper et al. 1978). It is worth mentioning that SMR_*i*_ as well as PMR_*i*_ are confounded by health differences between occupational cohorts and the general population (Park et al. 1991; Roman et al. 1984). But what happens when a large share of the deaths is caused by an occupational disease? The answer is clear: Then, the PMRs for all other diseases are not really an appropriate estimate of the corresponding risk. A more appropriate solution could be found by excluding the deaths due to the competing cause from the calculation in the cohort as well as from the reference population, i.e., from the expected values. Therefore, the adjusted estimate for the PMR_*i*_ can be derived by

2 $${\text{PMR}}_{i}^{*} = \frac{{\frac{{{\text{OBS}}_{i} }}{{({\text{OBS}} - {\text{OBS}}_{0} )}}}}{{\frac{{{\text{EXP}}_{i} }}{{({\text{EXP}} - {\text{EXP}}_{0} )}}}}$$

Combining Eqs. 1 and 2, the adjusted estimate of the cause-specific SMR can then be derived by

3 $${\text{SMR}}_{i}^{*} = {\text{PMR}}_{i}^{*} \times {\text{SMR}} = \frac{{\frac{{{\text{OBS}}_{i} }}{{({\text{OBS}} - {\text{OBS}}_{0} )}}}}{{\frac{{{\text{EXP}}_{i} }}{{({\text{EXP}} - {\text{EXP}}_{0} )}}}} \times \frac{\text{OBS}}{\text{EXP}}$$

Hence, the estimate can be rewritten in the usual form:

4 $${\text{SMR}}_{i}^{*} = \frac{{{\text{OBS}}_{i} }}{{{\text{EXP}}_{i}^{*} }}$$

where

5 $${\text{EXP}}_{i}^{*} = {\text{EXP}}_{i} \times \frac{{{\text{EXP}} \times ({\text{OBS}} - {\text{OBS}}_{0} )}}{{({\text{EXP}} - {\text{EXP}}_{0} ) \times {\text{OBS}}}}$$

EXP_0_, the number of expected deaths in the cohort due to the assumed occupational disease and calculated on the basis of population-based reference rates, is very small in comparison with the number of overall expected deaths. Thus, it approximately holds

6 $${\text{EXP}}_{i}^{*} \approx {\text{EXP}}_{i} \times \frac{{({\text{OBS}} - {\text{OBS}}_{0} )}}{\text{OBS}}$$

If one applies the approach to the data published for those miners suffering from progressive massive fibrosis (PMF) in the US cohort of coal miners (Attfield and Kuempel 2008), one receives for the expected number of lung cancer deaths

$${\text{EXP}}_{\text{LC}} = \frac{7}{0.69} = 10.14$$

and, applying formula 5,

$${\text{EXP}}_{\text{LC}}^{*} = \frac{7}{0.69} \times \frac{100 \times (137 - 67)}{(100 - 4.4) \times 137} = 5.42$$

Hence, the adjusted SMR for lung cancer is calculated to be:

$${\text{SMR}}_{\text{LC}}^{*} = \, 1.29\, [0.52 \, {-} \, 2.66 ]_{95\,\% } .$$

## Results

Applying the above-developed method to the US coal miner cohort (Attfield and Kuempel 2008), the difference between the original SMR and the adjusted SMR increases with the severity of CWP (Table 2). Similar results (Table 3) are derived for the Rhondda Fach cohort (Atuhaire et al. 1985). The highest estimates for lung cancer risk are derived for the most severe forms of CWP.

**Table 2:** Recalculation of SMR for lung cancer in the US coal miner cohort (Attfield and Kuempel 2008)

Subcohort	OBS	EXP	SMR	EXP*	SMR*	95 % CI
*By CWP status*						
CWP, Cat 0	294	260.18	1.13	246.18	1.19	1.06–1.34
CWP, Cat 1	23	25.84	0.89	23.45	0.98	0.62–1.47
CWP, Cat 2	6	11.32	0.53	8.01	0.75	0.27–1.63
CWP, Cat 3	1	3.03	0.33	1.80	0.56	0.01–3.10
PMF	7	10.14	0.69	5.42	1.29	0.52–2.66
*By region*						
Anthracite	18	22.50	0.80	11.85	1.52	0.90–2.40
East Appalachia	43	44.79	0.96	40.46	1.06	0.77–1.43
West Appalachia	195	166.67	1.17	161.56	1.21	1.04–1.39
Mid-west	58	40.00	1.45	39.06	1.49	1.13–1.92
West	17	36.17	0.47	32.24	0.53	0.31–0.84
Total cohort	331	309.35	1.07	286.00	1.16	1.04–1.29

**Table 3:** Recalculation of SMR for lung cancer in the Rhondda Fach cohort (Atuhaire et al. 1985)

CWP category	OBS	EXP	SMR	EXP*	SMR*	95 % CI
Cat 0	100	130.72	0.77	130.12	0.77	0.62–0.94
Cat 1	22	35.09	0.63	34.85	0.63	0.40–0.96
Cat 2	25	27.32	0.92	26.03	0.96	0.62–1.42
Cat 3	13	15.15	0.86	13.83	0.94	0.50–1.61
A	12	17.44	0.69	14.47	0.83	0.43–1.45
B and C	19	20.97	0.91	10.98	1.73	1.04–2.70

The consideration of CWP leads to an increase in the corresponding SMR for lung cancer (Table 4) in other cohort studies, too. Again, the strongest increase is observed for workers with CWP (Meijers et al. 1991; Starzynski et al. 1996b). Starzynski and colleagues have also tried to differentiate between miners with CWP by a surrogate of coal dust concentration—placing miners into three groups (Starzynski et al. 1996a). The original calculated SMR for lung cancer is negatively correlated with this measure, but the adjusted SMRs are constant over those three groups (data not shown).

**Table 4:** Recalculation of SMR for lung cancer in cohorts of coal workers

Study	Subcohort	OBS	EXP	SMR	EXP*	SMR*	95 % CI
Meijers et al. (1991)	Workers with CWP	19	14.50	1.31	12.63	1.50	0.91–2.35
Starzynski et al. (1996a, b)	Workers with CWP	153	146.73	1.04	122.13	1.25	1.06–1.47
Miller and MacCalman (2010)	1959–1974	160	204.34	0.78	196.16	0.82	0.69–0.95
Miller and MacCalman (2010)	1975–1989	403	425.55	0.95	394.79	1.02	0.92–1.13
Miller and MacCalman (2010)	1990–2005	395	341.40	1.16	324.49	1.22	1.10–1.34
Miller and MacCalman (2010)	1959–2005	958	1034.56	0.93	975.29	0.98	0.92–1.05
Graber et al. (2014)	1969–2007	568	524.00	1.08	488.67	1.16	1.07–1.26

The presented method can be applied analogously to data from cohort studies of workers occupationally exposed to respirable silica (quartz) or even cohorts of silicotic patients. Results concerning lung cancer for some cohort studies among workers compensated for silicosis are given in Table 5 (Carta et al. 2001; Ebihara and Kawami 1998; Marinaccio et al. 2006; Scarselli et al. 2011; Starzynski et al. 1996b). The applicability of this method is not restricted to lung cancer as the outcome of interest. The method also leads to a relevant increase in the risk estimates for other outcomes such as ischemic heart disease as demonstrated for the study on Rhondda Fach miners in Table 6 (Atuhaire et al. 1986). The approach could also be used outside of occupational epidemiology, for example, in the analysis of mortality in tuberculosis patients (Davis et al. 1989).

**Table 5:** Recalculation of SMR for lung cancer among silicotic patients

Study	Subcohort	OBS	EXP	SMR	EXP*	SMR*	95 % CI
Starzynski et al. (1996a, b)	Foundry workers	69	43.39	1.59	36.13	1.91	1.49–2.42
Ebihara and Kawami (1998)		51	15.42	3.31	7.21	7.07	5.27–9.30
Carta et al. (2001)		34	24.90	1.37	12.96	2.62	1.82–3.67
Marinaccio et al. (2006)	Males	798.06	723.88	1.10	609.00	1.31	1.22–1.40
Marinaccio et al. (2006)	Females	6.06	6.16	1.07	5.61	1.18	0.46–2.47
Scarselli et al. (2011)		138.78	100.04	1.39	83.21	1.67	1.40–1.97

**Table 6:** Recalculation of SMR for some specific causes in Rhondda Fach by category of CWP (Atuhaire et al. 1985)

Category of CWP	0–3		A		B and C	
Cause of death	SMR	SMR*	SMR	SMR*	SMR	SMR*
All cancers	0.90	0.91	0.98	1.18	0.99	1.89
Gastric cancer	1.42	1.44	2.17	2.61	1.51	2.89
Lung cancer	0.77	0.78	0.69	0.83	0.91	1.73
All circulatory	1.17	1.19	0.90	1.08	0.84	1.60
Ischemic heart disease	1.16	1.18	0.83	1.01	0.88	1.68

## Discussion

Coal miners are exposed to a number of lung carcinogens, particularly coal mine dust, which contains respirable silica (quartz) to a varying degree. Earlier cohort mortality studies of coal miners suggested that coal mining may even be protective for lung cancer (Goldman 1965). But their analyses were based on commonly calculated SMRs using population-based mortality rates as reference. This method is not appropriate, if one investigates subgroups of miners suffering from CWP with respect to other diseases such as lung cancer or heart diseases, because advanced CWP can be fatal and the corresponding excess mortality to CWP is not reflected in population-based mortality rates. Hence, the mortality from causes other than CWP is underestimated by the standard methods in such (nonstandard) cohorts. Moreover, the higher the severity of CWP, the more pronounced this underestimation will be.

Of course, the proposed method is only a crude approximation. A better (albeit theoretical) approach would be to estimate the age-specific mortality rates for CWP from the cohort under investigation and integrate them into the set of population-based reference rates. Such an approach would lead in the notations of Table 1 to SMR_0_ = 1 and an increase in all other cause-specific SMRs in comparison with the standard approach. However, in practice, such a comparison of subgroups defined by different severity of CWP would be difficult because of sparse data for the estimation of age-specific mortality rates for CWP stratified by these subgroups. Needless to say, such an approach is applicable only if the case-wise data records of the whole cohort are available.

In light of these considerations, a practicable approach could be to require ${\text{SMR}}_{0}^{*} = 1$ generally, i.e., to assume that the (adjusted) number of expected cases of deaths from the competing cause is equal to the number of observed cases. This assumption can be rewritten as

7 $${\text{EXP}}_{0}^{*} = {\text{OBS}}_{0} .$$

Hence, the number of expected cases for all other causes of death must be proportionally reduced by a factor *f* to not change the overall SMR, i.e.,

8 $${\text{EXP}}_{i}^{*} = {\text{EXP}}_{i} \times f$$

where

9 $$f = \frac{{{\text{EXP}} - {\text{EXP}}_{0}^{*} }}{{{\text{EXP}} - {\text{EXP}}_{0} }} = \frac{{{\text{EXP}} - {\text{OBS}}_{0} }}{{{\text{EXP}} - {\text{EXP}}_{0} }}$$

At first glance, this seems to be a natural approach to adjust for CWP or another occupational disease as competing cause of death. This approach would almost always lead to higher SMRs in comparison with the initially described ones. But in some studies the number of observed deaths from the occupational disease exceeds even the total number of expected deaths for the whole cohort, as for example in the study of Ebihara and Kawami (1998). Hence, the adjusted number of expected deaths from lung cancer would have to be negative under such circumstances. Therefore, Eq. 7 is not an assumption to derive a suitable approximation procedure, especially if the overall SMR is considerably elevated.

A further approach would be to require ${\text{SMR}}_{0}^{*} = {\text{SMR}}$ generally, i.e., to assume that the (adjusted) SMR for the competing cause of death is equal to the overall SMR. This assumption can be rewritten as

10 $$\frac{{{\text{OBS}}_{0} }}{{{\text{EXP}}_{0}^{*} }} = \frac{\text{OBS}}{\text{EXP}}$$

As in the previously described approach, the number of expected cases for all other causes of death must be proportionally reduced by the factor *f*, where now holds

11 $$f = \frac{{{\text{EXP}} - {\text{EXP}} \times \frac{{{\text{OBS}}_{0} }}{\text{OBS}}}}{{{\text{EXP}} - {\text{EXP}}_{0} }} = \frac{{{\text{EXP}} \times ({\text{OBS}} - {\text{OBS}}_{0} )}}{{({\text{EXP}} - {\text{EXP}}_{0} ) \times {\text{OBS}}}}$$

It follows that factor *f* is identical to the factor derived in Eq. 5. Therefore, the proposed method to adjust the SMRs for competing causes of death is equivalent to assuming equality between the overall SMR and the adjusted SMR for the competing cause of death ${\text{SMR}}_{0}^{*} = {\text{SMR}}$. This assumption seems reasonable given that the mortality in a diseased occupational cohort is being investigated. Besides the usually used criterion concerning the occupational exposure, a further selection criterion is a medically confirmed occupational disease like CWP or silicosis, for which a causal link to the occupational exposure is unequivocally established. If the occupational disease itself could lead to death and, hence, could be documented as underlying cause of death on the death certificate, then the use of population-based reference rate for the calculation of SMRs in the standard way is improper, because the population-based reference rates do not take into consideration the excess mortality due to the occupational disease as an underlying cause of death.

The proposed approach to adjust SMRs for competing causes of death can be compared to a certain degree to the so-called Axelson method, which is an indirect method for assessing the effects of tobacco use in occupational studies (Axelson 1978; Axelson and Steenland 1988; Checkoway and Waldman 1985).

Wong and Decouflé have shown that the validity of the ordinary PMR depends on the homogeneity of the age-specific overall SMR (Wong and Decoufle 1982). Therefore, applying the adjustment approach described above, one should take into account the possible heterogeneity in the age-specific SMRs. However, most occupational cohort studies’ results are stratified by exposure groups only. A further stratification by age is mostly missing due to sparse case numbers. In case of known age-specific heterogeneity, the method can be applied separately for each age group to overcome this problem.

It is worth mentioning that even if the described approach reduces the bias due to improper reference rates for the calculation of SMRs, other types of biases may seriously influence the results. For example, in cohort studies, on coal miners suffering from CWP, a possible diagnostic bias as well as a possible reporting bias should be taken into account. As already noted by James, the diagnosis of coexisting lung cancer by X-ray is difficult or impossible, especially in those miners with massive pneumoconiosis (James 1955). Moreover, knowledge of the radiographic category of CWP may have influenced a physician’s decision to document pneumoconiosis as the underlying cause of death (Kuempel et al. 1995). Therefore, lung cancer could be underrepresented as underlying cause of death.

Finally, it should be noted that in cases where adequate incidence data are available for the reference population, the analysis of standardized incidence ratios (SIRs) should be preferred over the SMR analysis. An adjustment for competing causes of death is not necessary when using SIRs, at least if the age-specific risks of the two diseases of interest are independent. Moreover, in contrast to a corresponding SMR analysis, reporting biases need also not be taken into account.

## References

[CR1] Attfield MD, Kuempel ED (2008). Mortality among US underground coal miners: a 23-year follow-up. Am J Ind Med.

[CR2] Atuhaire LK, Campbell MJ, Cochrane AL, Jones M, Moore F (1985). Mortality of men in the Rhondda Fach 1950–80. Br J Ind Med.

[CR3] Atuhaire LK, Campbell MJ, Cochane AL, Jones M, Moore F (1986). Specific causes of death in miners and ex-miners of the Rhondda Fach 1950–80. Br J Ind Med.

[CR4] Axelson O (1978). Aspects on confounding in occupational health epidemiology. Scand J Work Environ Health.

[CR5] Axelson O, Steenland K (1988). Indirect methods of assessing the effects of tobacco use in occupational studies. Am J Ind Med.

[CR6] Carta P, Aru G, Manca P (2001). Mortality from lung cancer among silicotic patients in Sardinia: an update study with 10 more years of follow up. Occup Environ Med.

[CR7] Checkoway H, Waldman GT (1985). Assessing the possible extent of confounding in occupational case-referent studies. Scand J Work Environ Health.

[CR8] Davis FG, Boice JD, Hrubec Z, Monson RR (1989). Cancer mortality in a radiation-exposed cohort of Massachusetts tuberculosis patients. Cancer Res.

[CR9] Decouflé P, Thomas TL, Pickle LW (1980). Comparison of the proportionate mortality ratio and standardized mortality ratio risk measures. Am J Epidemiol.

[CR10] Ebihara I, Kawami M (1998). Lung cancer and immunopathologic diseases among copper miners in a small copper mine, stone masons and pneumoconiotic patients in Japan. J Sci Labour.

[CR11] Goldman KP (1965). Mortality of coal-miners from carcinoma of the lung. Br J Ind Med.

[CR011] Graber JM, Stayner LT, Cohen RA, Conroy LM, Attfield MD (2014). Respiratory disease mortality among US coal miners; results after 37 years of follow-up. Occup Environ Med.

[CR12] James WR (1955). Primary lung cancer in South Wales coal-workers with pneumoconiosis. Br J Ind Med.

[CR13] Kuempel ED, Stayner LT, Attfield MD, Buncher CR (1995). Exposure-response analysis of mortality among coal miners in the United States. Am J Ind Med.

[CR14] Kupper LL, McMichael AJ, Symons MJ, Most BM (1978). On the utility of proportional mortality analysis. J Chronic Dis.

[CR15] Marinaccio A (2006). Retrospective mortality cohort study of Italian workers compensated for silicosis. Occup Environ Med.

[CR16] Meijers JM, Swaen GM, Slangen JJ, van Vliet K, Sturmans F (1991). Long-term mortality in miners with coal workers’ pneumoconiosis in The Netherlands: a pilot study. Am J Ind Med.

[CR17] Miller BG, MacCalman L (2010). Cause-specific mortality in British coal workers and exposure to respirable dust and quartz. Occup Environ Med.

[CR18] Morfeld P, Stegmaier C, Ziegler H, Emmerich M (2005) Krebsmorbidität und Krebsmortalität saarländischer Steinkohlenbergleute in Abhängigkeit von Staubexposition und Pneumokonioseentwicklung (Phase IV). Report from Arbeitsgemeinschaft des Saarlandes zur Erforschung und Förderung des Gesundheitsschutzes im Bergbau e. V. (AGiB). Verlag Alma Mater. Saarbrücken, p 107

[CR19] Park RM, Maizlish NA, Punnett L, Moure-Eraso R, Silverstein MA (1991). A comparison of PMRs and SMRs as estimators of occupational mortality. Epidemiology.

[CR20] Roman E, Beral V, Inskip H, McDowall M, Adelstein A (1984). A comparison of standardized and proportional mortality ratios. Stat Med.

[CR21] Scarselli A, Binazzi A, Forastiere F, Cavariani F, Marinaccio A (2011). Industry and job-specific mortality after occupational exposure to silica dust. Occup Med (Lond).

[CR22] Starzynski Z, Marek K, Kujawska A, Szymczak W (1996). Mortality among coal miners with pneumoconiosis in Poland. Int J Occup Med Environ Health.

[CR23] Starzynski Z, Marek K, Kujawska A, Szymczak W (1996). Mortality among different occupational groups of workers with pneumoconiosis: results from a register-based cohort study. Am J Ind Med.

[CR24] Tomaskova H, Jirak Z, Splichalova A, Urban P (2012). Cancer incidence in Czech black coal miners in association with coalworkers’ pneumoconiosis. Int J Occup Med Environ Health.

[CR25] Wong O, Decoufle P (1982). Methodological issues involving the standardized mortality ratio and proportionate mortality ratio in occupational studies. J Occup Med.

